# AI and the future of teachers: predicting stakeholders’ expectations of teacher job loss from AI-related institutional conditions

**DOI:** 10.3389/frai.2026.1933015

**Published:** 2026-09-30

**Authors:** Georgios P. Georgiou

**Affiliations:** Department of Languages and Literature, University of Nicosia, Nicosia, Cyprus

**Keywords:** artificial intelligence, institutional indicators, job loss, machine learning, prediction, teachers

## Abstract

**Introduction:**

Artificial intelligence (AI) is transforming educational work, although claims that it will replace teachers often overlook the institutional conditions that make teacher job loss appear more or less likely. This study examined whether current AI-related institutional risk conditions predict stakeholder expectations of teacher job loss over the next decade.

**Methods:**

A purposive sample of 300 education and AI stakeholders rated the extent to which 11 conditions were currently present and provided a separate 0–10 score indicating the extent to which they expected AI to contribute to teacher job loss.

**Results:**

Overall job-loss expectations were low to moderate, and the institutional AI condition indicators showed good internal consistency. The machine learning model showed good predictive performance in the original held-out test set (*R*^2^ = 0.683) and stable performance across 100 repeated train-test splits (mean *R*^2^ = 0.598). The explainable AI analysis showed that the strongest contribution band consisted of automated feedback, large-scale AI adoption, and standardized content delivery.

**Discussion:**

The findings suggest that the extent to which stakeholders perceive AI-related institutional risk conditions to be currently present in educational settings is associated with the extent of teacher job loss they expect. They further indicate that this risk is not an inevitable technological outcome, but is embedded in a broader institutional ecology, with expectations clustering more strongly around direct forms of AI-mediated instructional restructuring than around indirect institutional or governance-related vulnerabilities.

## 1 Introduction

Artificial intelligence (AI) is increasingly reshaping the organization of educational work. What began as a set of experimental technologies has developed into a wider ecosystem of adaptive learning platforms, intelligent tutoring systems, automated assessment tools, generative writing systems, and learning analytics dashboards that are now becoming more visible in educational institutions (Georgiou, 2026a). Survey data from the 2023-2024 school year show that approximately one quarter of United States teachers reported using AI tools for instructional planning or teaching, while nearly 60% of school principals reported using AI tools for their own work (Kaufman et al., 2025). These developments are occurring within a broader labor-market context in which AI is expected to transform work substantially. The World Economic Forum’s Future of Jobs Report 2025 projects that AI will displace 92 million jobs globally while creating 170 million new ones (Leopold et al., 2025). Although education is often framed as a human-centered profession, it is not insulated from these technological and institutional pressures.

As AI systems become more capable in content generation, assessment, feedback, student profiling, and administrative support, teachers increasingly express concerns about job displacement, reduced creativity and critical thinking, unintended consequences, and trust in AI’s reliability (Gârdan et al., 2025). However, concerns about AI-related teacher job loss should not be understood simply as a belief that machines will replace teachers wholesale. Empirical and review-based research suggests that teachers’ fears are usually embedded in wider concerns about professional autonomy, role change, institutional control, ethical risk, and preparedness. For example, Chan and Tsi’s (2024) mixed-methods study of 184 teachers and 399 students in Hong Kong found that some participants believed generative AI could eventually replace teachers, yet most emphasized that human teachers remain difficult to replace because of critical thinking, emotions, social interaction, and social–emotional competencies. Similarly, a survey of 453 Ukrainian university teachers by Okulicz-Kozaryn et al. (2026) found that respondents did not expect large-scale AI replacement within 5 years, although their fear of losing control over AI technologies was stronger than their fear of job displacement. Broader evidence also suggests that educator resistance to AI is shaped by job-displacement worries, role ambiguity, technological complexity, insufficient training, and ethical concerns (McGehee, 2024). Recent qualitative work with in-service teachers further shows that AI is often viewed as useful for efficiency, assessment, access to knowledge, and lesson support, while also raising concerns about emotional detachment, weakened communication skills, technological dependency, and the need for structured professional development (Kayıran et al., 2026).

Debates about whether AI will “replace teachers” therefore rely on an overly binary framing. AI is often presented either as a transformative support for teachers or as an existential threat to the profession (Day, 2026). This framing obscures a more important issue: perceived AI-related teacher job loss is unlikely to arise from AI tools alone, but from the institutional conditions under which such tools are adopted, governed, and embedded in educational work. Teacher job-loss expectations should therefore be distinguished from broader forms of teacher displacement. Displacement may involve not only the reduction of teaching positions, but also the automation of specific tasks, the standardization of instructional practice through digital platforms, the erosion of professional discretion, the expansion of surveillance and data-management roles, and the gradual narrowing of teachers’ work under institutional cost-cutting pressures. In such contexts, teachers may remain physically present in educational systems while their roles are reorganized around platform supervision, exception handling, and data interpretation. Recent scholarship has warned that as AI systems take over instructional and assessment functions, educators may shift from pedagogical leaders to data custodians who mediate between algorithmic systems and learners rather than authoring instruction themselves (Ambady and Thomas, 2026).

The purpose of the present study is to examine whether the perceived current institutional AI-related risk conditions can predict stakeholder-estimated teacher job-loss expectations over the next 10 years. Rather than asking participants only whether they personally use AI tools, the study focuses on the extent to which specific institutional, technological, pedagogical, and governance conditions are currently visible in educational contexts familiar to them. This design allows the study to move beyond general speculation about AI and the potential loss of teaching jobs by testing whether present-day perceived institutional conditions contain a meaningful predictive signal for stakeholder-estimated future job-loss expectations. The study is grounded in four interconnected theoretical perspectives: task-based automation theory, platformization and datafication, teacher agency and AI literacy, and human-relational pedagogy. Together, these perspectives explain why AI-related teacher job-loss expectations should be conceptualized not as a direct technological outcome, but as a perceived labor-market risk understood in relation to the interaction between what AI systems can automate, how institutions adopt them, how teachers are prepared to work with them, and how strongly education systems protect the relational and professional dimensions of teaching.

Task-based automation theory provides the first lens. Autor (2015) argued that computers substitute most readily for routine, codifiable tasks while complementing forms of work that require problem-solving, adaptability, and creativity. This shifts the displacement question from whether entire occupations will disappear to which tasks within occupations are most vulnerable to automation. Empirical work by Acemoglu and Restrepo (2020) similarly shows that automation technologies tend to displace workers in occupations or tasks characterized by repetition and codifiability. Subsequent scholarship distinguishes between substitution, where automation transfers tasks from humans to machines, and augmentation, where technology extends human capabilities (Biström and Mollwing, 2026). Applied to education, this perspective suggests that AI is more likely to affect grading, quiz generation, attendance tracking, progress monitoring, feedback drafting, and administrative reporting before it replaces the more relational, ethical, and context-sensitive dimensions of teaching. Systematic reviews of AI in higher education identify key domains such as profiling and prediction, assessment and evaluation, adaptive systems and personalization, and intelligent tutoring systems (Zawacki-Richter et al., 2019), which are precisely the areas where teaching can be decomposed into data-rich and machine-readable tasks. More recent conceptual work argues that while AI may reduce cognitive load and support complex instructional tasks, it may also displace teacher judgment and weaken reflective professional expertise (Rind, 2026).

Platformization and datafication provide the second theoretical lens. Williamson (2017) showed how large-scale educational platforms generate extensive data from student–software interactions, enabling predictive analytics about engagement, performance, and learning behavior. Such analytics may support teachers, but they may also substitute for teacher judgment when instructional decisions are increasingly mediated by algorithmic recommendations. Williamson et al. (2020) further argue that datafication can reshape what counts as teaching and learning by privileging what can be measured, predicted, and optimized by digital systems. As educational platforms expand, pedagogical authority may shift away from teachers and toward corporate infrastructures, standardized dashboards, and algorithmically structured curricula. The growing influence of large technology companies in education, therefore, raises concerns about institutional autonomy, teacher discretion, and the values embedded in platform-mediated learning environments (Rind, 2026). When AI platforms are adopted at scale, the key risk is not only automation but also pedagogical deskilling through disuse: if teachers are less involved in core instructional decisions, the professional knowledge sustained through those decisions may gradually erode (Biström and Mollwing, 2026). This risk may be amplified by institutional cost pressures, especially in contexts characterized by standardization and scalable content delivery. As AI and robotics automate repetitive tasks, some roles may be displaced while new roles in data analytics, AI ethics, and system governance emerge (Leopold et al., 2025). In education, however, the promise of efficiency may encourage institutions to reduce reliance on human instructional labor.

Teacher agency and AI literacy form the third theoretical dimension. The UNESCO AI Competency Framework for Teachers emphasizes a human-centered approach to AI integration, linking teacher AI competencies to human rights, accountability, and responsible governance across five domains (Miao and Cukurova, 2024). The Beijing Consensus on AI and Education similarly states that teachers cannot be displaced by machines, while also emphasizing the need to strengthen AI literacy across society and prepare teachers to work effectively in AI-rich environments (UNESCO, 2019). Yet the expansion of intelligent tutoring systems and AI-driven educational tools has also been associated with concerns about the automation of teaching roles, reduced professional autonomy, and the weakening of traditional educator expertise (Daher, 2025). Research with pre-service teachers suggests that concerns about automation-driven job loss and diminished human agency may persist even after direct engagement with AI technologies, indicating that such anxiety reflects structural concerns rather than simple unfamiliarity (Sat, 2025). Teacher autonomy is therefore central. Teachers with stronger autonomy are better positioned to adapt and innovate as AI tools enter practice, and autonomy can strengthen teachers’ sense of ownership over AI integration (Zhao and Huang, 2025). Conversely, weak AI literacy and insufficient institutional support may increase vulnerability to platform dependency and reduce educators’ ability to critically evaluate automated recommendations. Selwyn (2024) further cautions that AI in education is limited by the restricted ways educational processes can be statistically modeled, the risk of reproducing social harms for minoritized students, the losses associated with making education more machine-readable, and the ecological costs of data-intensive systems.

Human-relational pedagogy provides the fourth and counterbalancing lens. Teaching is not only a technical activity of delivering content, assessing performance, or managing data. It is also interpretive, ethical, relational, and grounded in professional judgment. Teaching depends on human cognition, motivation, social interaction, emotional responsiveness, and contextual understanding in ways that cannot be fully specified or exhaustively modeled by AI systems (Han, 2026). Studies of teacher and student perceptions consistently suggest that many participants view human teachers as irreplaceable because of their creativity, critical thinking, emotional responsiveness, and role in supporting social–emotional development (Chan and Tsi, 2024). Scenario analyses of AI in education distinguish between labor-replacing classrooms, where AI tutors displace core instructional tasks and teachers are redeployed into surveillance and exception handling, and human–AI teaming, where AI is implemented as a teacher-governed support system designed to augment professional judgment (Biström and Mollwing, 2026). These scenarios illustrate that job-loss expectations are not determined by technology alone. They depend on whether AI is implemented to substitute for human teaching labor or to strengthen teacher agency and human-centered pedagogy.

Despite growing interest in AI and the future of teaching, three limitations remain in the existing literature. First, much of the debate continues to focus on broad claims about whether AI will or will not replace teachers, rather than identifying the institutional conditions under which stakeholders perceive teachers’ jobs to be at greater risk. Second, existing studies often examine AI adoption, attitudes, or ethical concerns without developing a focused measure of perceived institutional AI-related risk conditions that may be associated with teacher job-loss expectations. Third, relatively little empirical research has tested whether present-day institutional conditions can predict informed stakeholders’ judgments about the future loss of teaching jobs due to AI. The present study addresses these gaps by developing 11 theoretically derived institutional AI-related risk-condition indicators and testing whether the extent to which stakeholders perceived these conditions to be present predicted their separate ratings of expected AI-related teacher job loss over the next decade. The primary theoretical contribution of this study is to advance an institutional account of perceived AI-related teacher job-loss risk by shifting attention from whether AI can replace teachers to the institutional conditions under which stakeholders perceive teacher job loss as more likely. Explainable machine learning provides the methodological means for examining the relative predictive contributions of these theoretically derived conditions. Prediction is used in the statistical sense, referring to the estimation of perceived job-loss expectation scores from current institutional AI-related conditions.

The following research questions were addressed:

1. What are the descriptive and reliability properties of the institutional AI-related risk-condition indicators?
2. Can the current indicators predict perceived AI-related teacher job-loss expectation scores over the next 10 years?
3. Which of the indicators contribute most strongly to the machine-learning prediction of perceived teacher job-loss expectation scores?

## 2 Methodology

### 2.1 Participants

The sample included 300 stakeholders from seven professional groups: school teachers (K–12) (*n* = 80), university teachers (*n* = 50), school leaders (*n* = 45), teacher educators (*n* = 40), educational technology specialists (n = 35), AI-in-education researchers (*n* = 25), and AI developers working in educational contexts (*n* = 25). The term stakeholder is used here to refer to participants whose professional roles positioned them to provide informed judgments about AI-related changes in education; it does not imply equal technical expertise in AI across all groups. The sample was mainly based in Cyprus. Gender distribution was balanced, with 153 female and 147 male participants. Participants’ ages ranged from 24 to 63 years (*M* = 42.71, SD = 7.92), and years of professional experience ranged from 1 to 36 years (*M* = 16.18, SD = 7.97).

Participants were eligible for inclusion if they were aged 18 years or older and had current or recent professional experience in one of the target stakeholder categories. Because the study focused on informed judgments about AI-related institutional change in education, participants were also required to report professional familiarity with at least one of the following areas: education practice, educational leadership, teacher education, educational technology, AI in education, or AI development for educational settings. Participants who did not meet these criteria were excluded from the final dataset.

Potential participants were identified through professional networks, academic publications, institutional contacts, and education-technology or AI-in-education events. A total of 1,015 individuals were invited to participate, of whom 342 completed the questionnaire, corresponding to an initial completion rate of 33.7%. After screening, 42 responses were excluded before analysis because they did not meet the study’s inclusion criteria. The final analytic sample, therefore, consisted of 300 participants, corresponding to a usable response rate of 29.6%. Recruitment was guided by predetermined stakeholder-category targets to ensure that the final sample included perspectives from classroom teaching, higher education, school leadership, teacher education, educational technology, AI-in-education research, and AI development. These targets were used to support stakeholder diversity rather than to create a demographically representative sample. The sample should therefore be understood as purposive and analytically diverse rather than representative of the wider population of educators, educational leaders, or AI professionals.

### 2.2 Instrument

The measurement instrument consisted of 11 theoretically derived items measuring the perceived current institutional AI-related conditions potentially associated with future teacher job-loss expectations. The items asked participants to rate the extent to which each condition was currently present in the educational context with which they were most familiar, including schools, universities, educational platforms, policy environments, and institutional governance structures. Each item was rated on a 5-point scale: 1 = not present, 2 = rarely present, 3 = emerging or present in limited settings, 4 = moderately present or increasingly common, and 5 = widely present or strongly established. Higher scores indicated stronger perceived current institutional, technological, pedagogical, or governance conditions that may make future teacher job loss appear more likely. Although the indicators differed in substantive framing, with some describing the presence of AI-related capabilities and others describing institutional deficits, all were coded in the same risk direction; therefore, no reverse-scoring was applied.

In addition to the 11 indicators of AI-related institutional risk conditions, participants provided a separate score reflecting their expectations of teacher job loss. This outcome variable measured the extent to which participants believed AI might contribute to the loss of teaching jobs over the next 10 years. Scores ranged from 0 to 10, with 0 indicating no expected job loss and 10 indicating a very high level of expected job loss. The scale was used because the outcome was conceptualized as a global expectancy judgment rather than as a multi-component attitude scale. This format allowed participants to provide a direct estimate of perceived AI-related teacher job-loss expectation while keeping the outcome analytically distinct from the 11 institutional condition indicators. This score was treated as the target variable in the machine-learning analysis and was conceptually distinct from the 11 predictor items. The outcome measured stakeholder expectations of possible future job loss, not actual teacher employment change.

The 11 indicators were developed through a theory-driven item-generation process. Literature on task-based automation, platformization and datafication, teacher agency and AI literacy, institutional governance, and human-relational pedagogy was reviewed to identify institutional conditions under which AI may reduce reliance on human instructional labor. The resulting items covered task-level automation, platform-mediated standardization, institutional and policy vulnerability, and human-relational dimensions of teaching. The indicators were treated as theoretically informed predictive variables representing distinct but related institutional conditions, rather than as reflective indicators of a single latent construct. Accordingly, internal consistency coefficients were reported as descriptive evidence of the indicators’ overall interrelatedness, not as evidence that they form a unidimensional psychometric scale.

To support content coverage, items were reviewed for theoretical coverage, clarity, and redundancy. Items were retained when they represented a distinct AI-related institutional condition that could plausibly be observed or judged by education and technology stakeholders. Items that overlapped conceptually were merged or reworded, and items that referred too directly to teacher job loss were avoided to reduce criterion contamination. The 11 items, together with the exact wording used in the questionnaire, are shown in Table 1.

**Table 1:** Full wording of the 11 institutional AI-related risk-condition indicators.

No.	Item abbreviation	Item wording
1	Routine repetitive tasks	AI tools are currently used, piloted, or institutionally discussed for supporting routine and repetitive teaching-related tasks.
2	Automated feedback	AI systems are currently used, piloted, or institutionally discussed for generating, supporting, or managing assessment and feedback processes.
3	Standardized content delivery	Educational institutions are currently showing increased reliance on standardized or content-delivery-heavy instruction supported by digital or AI-mediated tools.
4	Low emotional support	Educational settings currently give limited institutional priority to human relational, emotional, or pastoral support in teaching.
5	Weak AI literacy	Educators currently show limited AI literacy or insufficient preparation for critically evaluating and using AI systems in educational contexts.
6	Cost-cutting pressure	Educational institutions currently face cost-cutting pressures that encourage scalable, efficiency-oriented, or AI-mediated instructional models.
7	Large-scale AI adoption	AI-powered educational platforms are currently being adopted, piloted, or considered at the institutional or system level.
8	Limited diversification	Educational institutions currently provide limited opportunities for teachers to take on new AI-related professional roles.
9	Scripted curricula	Educational systems are currently showing increased reliance on AI-scripted, AI-standardized, or AI-generated curriculum materials.
10	Weak policy safeguards	Current policy protections, institutional guidelines, or accountability mechanisms for responsible AI use in education remain limited or insufficient.
11	Low creativity	Educational institutions currently provide limited formal recognition for teacher creativity, professional judgment, and pastoral care when adopting AI-mediated teaching tools.

### 2.3 Procedure

Potential participants were informed about the study’s goal. After providing informed consent, participants completed a self-administered online questionnaire hosted on Google Forms. The questionnaire first gathered demographic and professional information, followed by the 11 indicators and the separate 0–10 teacher job-loss expectation score. Participants were asked to rate the extent to which each condition was currently present in the educational context they knew best. They were then asked to “estimate the extent to which AI might contribute to the loss of teaching jobs over the next 10 years”. The questionnaire was brief and designed to minimize respondent burden. Data collection took place over 1 month. All responses were anonymous and stored in password-protected digital files. The final dataset was exported as an Excel file and subjected to the analysis described below.

### 2.4 Data analysis

Data were analyzed in R (R Core Team, 2026). First, descriptive statistics were calculated for the 11 institutional conditions, including means, standard deviations, ranges, skewness, and kurtosis. These analyses were used to examine the distribution of item responses and to determine whether the items showed sufficient variability.

Internal consistency reliability was examined using Cronbach’s alpha (*α*) and McDonald’s omega total (*ω*), and corrected item-total correlations were calculated to assess whether the indicators showed sufficient coherence as a theoretically derived predictor set. After the descriptive and reliability analyses were completed, a supervised machine learning analysis was conducted to predict the perceived teacher job-loss expectation score. The 11 indicators were used as predictors, and the independently rated future teacher job-loss expectation score was used as the target variable. A LightGBM regression model (Ke et al., 2017) was trained using an 80–20 train-test split. Five-fold cross-validation was applied within the training set to identify the optimal number of boosting iterations. Final model performance was evaluated on the held-out test set using root mean squared error (RMSE), mean absolute error (MAE), and *R*^2^. Zero-order Pearson correlations were also calculated between each institutional indicator and the teacher job-loss expectation score to examine the bivariate predictor–outcome associations.

SHapley Additive exPlanations (SHAP)-based feature contribution analysis was conducted to interpret the relative contribution of each predictor to the model’s predictions (Lundberg and Lee, 2017). This allowed the study to identify which indicators contributed most strongly to predicted teacher job-loss expectations.

## 3 Results

### 3.1 Descriptive statistics of the institutional AI-related risk-condition indicators

Descriptive statistics were first calculated for the institutional indicators. No missing values were observed across the 11 items. Overall, item means ranged from low to moderately high levels on the five-point scale. All item responses ranged from 1 to 5, showing that the full response range was used for each indicator. Median values were 3 for weak AI literacy, weak policy safeguards, automated feedback, routine repetitive tasks, large-scale AI adoption, and cost-cutting pressure, and 2 for standardized content delivery, limited role diversification, scripted curricula, low creativity and judgment, and low emotional support. Skewness values ranged from −0.05 to 0.73, indicating approximately symmetric to moderately positively skewed distributions. Kurtosis values ranged from −0.77 to −0.48, suggesting relatively flat but acceptable distributional variation across items.

The highest-rated item was weak AI literacy (*M* = 3.25, SD = 1.03), followed by weak policy safeguards (*M* = 3.10, SD = 1.02), automated feedback (*M* = 2.95, SD = 1.00), routine repetitive tasks (*M* = 2.85, SD = 1.02), and large-scale AI adoption (*M* = 2.70, SD = 1.04). These results suggest that participants perceived these as the most visible AI-related institutional conditions. Cost-cutting pressure also showed a moderate level of perceived presence (*M* = 2.60, SD = 1.04). The lowest-rated items were low emotional support (*M* = 1.85, SD = 0.98), scripted curricula (*M* = 2.00, SD = 1.00), low creativity and judgment (*M* = 2.10, SD = 1.02), limited role diversification (*M* = 2.25, SD = 1.03), and standardized content delivery (*M* = 2.40, SD = 1.04). Figure 1 illustrates the mean ratings of the indicators.

**Figure 1:**
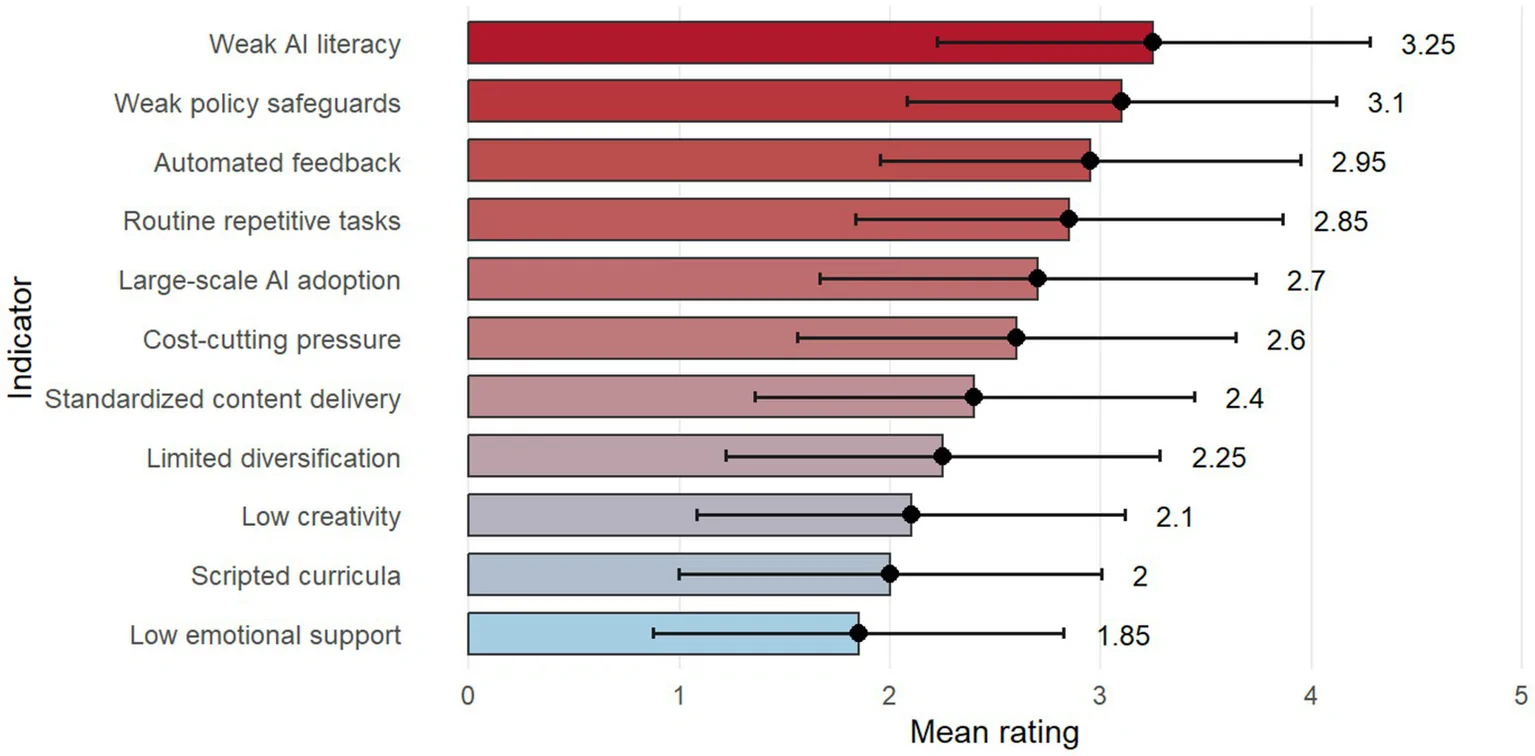
Mean ratings of institutional AI-related risk-condition indicators. Bars show the mean rating for each indicator on a 1–5 scale, where higher scores indicate greater perceived current AI-related risk conditions in educational settings. Error bars represent ±1 standard deviation.

Descriptive statistics were also calculated for the perceived teacher job-loss expectation score. The mean score was 3.27 (SD = 1.94), suggesting a low-to-moderate overall level of perceived AI-related teacher job-loss expectation. The distribution showed positive skewness (skewness = 0.79), indicating that lower job-loss expectation scores were more common than higher scores. Excess kurtosis was 0.45, suggesting a slightly more peaked distribution than a normal distribution.

An exploratory one-way ANOVA was conducted to examine whether perceived teacher job-loss expectation scores differed across stakeholder groups. The omnibus test was not statistically significant, *F*(6, 293) = 0.141, *p* = 0.991, *η*^2^ = 0.003. Assumption checks did not indicate major violations: the Shapiro–Wilk test of residuals was not statistically significant, *W* = 0.993, *p* = 0.166, and Levene’s test indicated homogeneity of variance, *F*(6, 293) = 2.108, *p* = 0.053. Tukey-adjusted posthoc comparisons also showed no statistically significant pairwise differences between stakeholder groups. These results suggest that perceived teacher job-loss expectations did not differ substantially by stakeholder category in the present sample.

### 3.2 Reliability of the institutional AI-related risk-condition indicators

The internal consistency of the institutional AI-related risk-condition indicators was good in the full sample. Cronbach’s alpha was *α* = 0.81, and McDonald’s omega total was similarly high, *ω* = 0.81. The average inter-item correlation was 0.28, indicating that the items were moderately related while still capturing distinguishable aspects of the risk conditions.

Corrected item-total correlations were all above the recommended threshold of 0.30. Item-rest correlations ranged from 0.42 for routine repetitive tasks, weak policy safeguards, and low creativity and judgment to 0.52 for automated feedback and cost-cutting pressure. The alpha-if-item-deleted values ranged from 0.79 to 0.80, and none exceeded the full-scale alpha. Therefore, deleting any item would not meaningfully improve internal consistency. Table 2 presents the item-level reliability diagnostics for the institutional AI-related risk-condition indicators.

**Table 2:** Item-level reliability diagnostics for the institutional AI-related risk-condition indicators.

Indicator	Corrected item-total correlation	Alpha if item deleted
Routine repetitive tasks	0.42	0.80
Automated feedback	0.52	0.79
Standardized content delivery	0.49	0.79
Low emotional support	0.44	0.80
Weak AI literacy	0.51	0.79
Cost-cutting pressure	0.52	0.79
Large-scale AI adoption	0.47	0.79
Limited role diversification	0.51	0.79
Scripted curricula	0.46	0.80
Weak policy safeguards	0.42	0.80
Low creativity and judgment	0.42	0.80

### 3.3 Machine-learning prediction of perceived teacher job-loss expectations

A supervised machine-learning analysis was conducted to examine whether the 11 institutional indicators could predict the perceived teacher job-loss expectation score. The target variable was the independently rated teacher job-loss expectation score, ranging from 0 to 10. The predictors were routine repetitive tasks, automated feedback, standardized content delivery, low emotional support, weak AI literacy, cost-cutting pressure, large-scale AI adoption, limited role diversification, scripted curricula, weak policy safeguards, and low creativity and judgment.

A LightGBM regression model was trained using an 80–20 train-test split. LightGBM was used as a flexible predictive model capable of accommodating complex relationships among the institutional indicators while also supporting SHAP-based feature interpretation. This combination allowed the analysis to evaluate predictive performance and examine the relative contribution of individual predictors within a unified modeling framework. The model used gradient boosting decision trees with the following hyperparameters: objective = regression, metric = RMSE, boosting = gbdt, learning rate = 0.01, number of leaves = 10, maximum depth = 4, feature fraction = 0.90, bagging fraction = 0.80, bagging frequency = 5, minimum data in leaf = 15, L1 regularization = 0.10, and L2 regularization = 0.50. Five-fold cross-validation was applied within the training set to identify the optimal number of boosting iterations, and the final model was then evaluated on the held-out test set.

Before interpreting the machine-learning model, diagnostic checks were conducted to examine whether the predictor set showed evidence of problematic multicollinearity or substantial common-method variance. Variance inflation factor values ranged from 1.23 to 1.43, remaining well below conventional thresholds for problematic multicollinearity (Kock, 2015). As a basic diagnostic check for common-method variance, Harman’s single-factor test was also conducted (Podsakoff et al., 2003). The first unrotated factor accounted for 37.75% of the total variance, below the commonly used 50% threshold. This suggests that the responses were not dominated by a single general method factor, although this test cannot rule out common-method influences.

The model demonstrated good predictive performance in the original held-out test set, with an RMSE of 1.028, an MAE of 0.814, and an *R*^2^ of 0.683. Figure 2 shows the correspondence between the observed and model-predicted teacher job-loss expectation scores in the held-out test set. To assess potential overfitting, performance was compared between the training and held-out test sets. The training RMSE was 0.912 compared with 1.028 on the test set, the training MAE was 0.709 compared with 0.814 on the test set, and the training *R*^2^ was 0.749 compared with 0.683 on the test set. These differences did not indicate substantial overfitting. This original held-out model was retained for the diagnostic, graphical, and SHAP-based explainability analyses.

To assess the stability of predictive performance across alternative data partitions, the 80–20 train-test procedure was additionally repeated across 100 random splits. Within each split, five-fold cross-validation was applied to the training set to identify the optimal number of boosting iterations, and model performance was then evaluated on the corresponding held-out test set. Across the 100 splits, the model achieved a mean RMSE of 1.136 (SD = 0.082), a mean MAE of 0.898 (SD = 0.070), and a mean *R*^2^ of 0.598 (SD = 0.063). These standard deviations represent split-to-split variability across alternative train-test partitions. The optimal number of boosting iterations averaged 411.85 (SD = 54.21). The original held-out split therefore showed stronger-than-average performance relative to the repeated-split distribution, while the repeated-split analysis indicated that predictive performance remained reasonably stable across alternative data partitions. Given that each held-out test set contained 60 respondents and the outcome was positively skewed, performance estimates from any single split may be sensitive to the allocation of relatively few high-scoring cases; this was one reason for examining performance across 100 alternative train-test partitions.

A regression tree was also estimated as a simple tree-based benchmark for LightGBM. This comparison was included because both models rely on decision-tree structures, while differing substantially in model complexity: the regression tree represents a single interpretable recursive-partitioning structure, whereas LightGBM combines an ensemble of boosted trees. The regression tree showed weaker predictive performance on the held-out test set (RMSE = 1.367, MAE = 1.068, *R*^2^ = 0.426) than LightGBM. This indicates that the boosted-tree approach provided substantially better predictive accuracy than the simpler single-tree specification. LightGBM was therefore retained as the primary predictive model and used for the subsequent SHAP-based feature contribution analysis.

**Figure 2:**
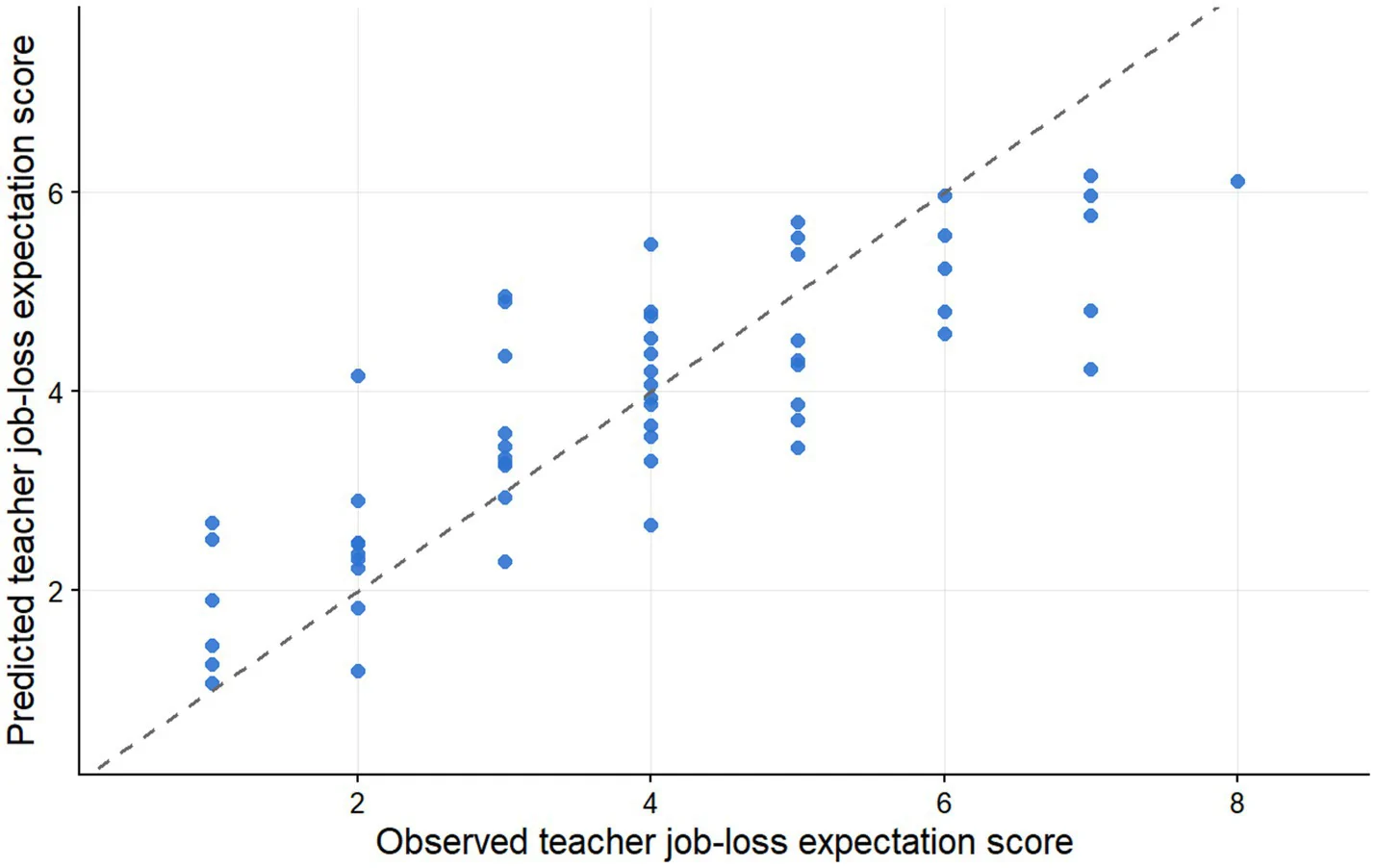
Observed versus predicted teacher job-loss expectation scores in the held-out test set. The scatterplot compares observed 0–10 teacher job-loss expectation scores with LightGBM-predicted scores. The dashed diagonal line represents perfect prediction, with points closer to the line indicating more accurate predictions.

Zero-order Pearson correlations were calculated between each institutional indicator and the teacher job-loss expectation score. All correlations were positive and statistically significant (all *p* < 0.001), ranging from *r* = 0.404 to *r* = 0.555. The strongest correlation was observed for automated feedback, followed by weak AI literacy, large-scale AI adoption, cost-cutting, and standardized content delivery. These bivariate associations provide a reference point for interpreting the subsequent SHAP results and assessing whether the model-based contribution pattern simply reflects the zero-order relationships with the outcome. Table 3 presents the zero-order correlations.

**Table 3:** Zero-order Pearson correlations between institutional AI-related risk-condition indicators and teacher job-loss expectation scores.

Institutional indicator	Pearson *r*	*p*
Automated feedback	0.555	<0.001
Weak AI literacy	0.525	<0.001
Large-scale AI adoption	0.525	<0.001
Cost-cutting pressure	0.525	<0.001
Standardized content delivery	0.517	<0.001
Limited diversification	0.498	<0.001
Routine repetitive tasks	0.453	<0.001
Scripted curricula	0.436	<0.001
Low creativity	0.419	<0.001
Low emotional support	0.417	<0.001
Weak policy safeguards	0.404	<0.001

To interpret the LightGBM model, SHAP feature contribution analysis was conducted. Mean absolute SHAP values were calculated for each predictor, with bootstrap 95% confidence intervals based on 2,000 resamples. The largest SHAP contribution was observed for automated feedback (mean |SHAP| = 0.345, 95% CI [0.304, 0.388]), followed by large-scale AI adoption (0.315, 95% CI [0.284, 0.347]) and standardized content delivery (0.305, 95% CI [0.265, 0.347]). Because the confidence intervals for these three predictors overlap, they should be interpreted as having broadly comparable contributions to the model rather than as clearly separable in rank order. Cost-cutting pressure also showed a meaningful contribution (0.224, 95% CI [0.198, 0.251]). A second group of predictors showed moderate contributions: limited diversification (0.190, 95% CI [0.160, 0.223]), routine repetitive tasks (0.179, 95% CI [0.143, 0.216]), low creativity and judgment (0.170, 95% CI [0.142, 0.199]), and low emotional support (0.168, 95% CI [0.146, 0.191]). The remaining predictors had smaller average contributions: scripted curricula (0.129, 95% CI [0.115, 0.142]), weak AI literacy (0.118, 95% CI [0.097, 0.139]), and weak policy safeguards (0.073, 95% CI [0.059, 0.087]) (see Figure 3). The SHAP contribution pattern did not fully mirror the zero-order correlation ordering. This divergence suggests that some indicators are strongly associated with job-loss expectations on their own but add less unique predictive information once the other institutional conditions are considered.

**Figure 3:**
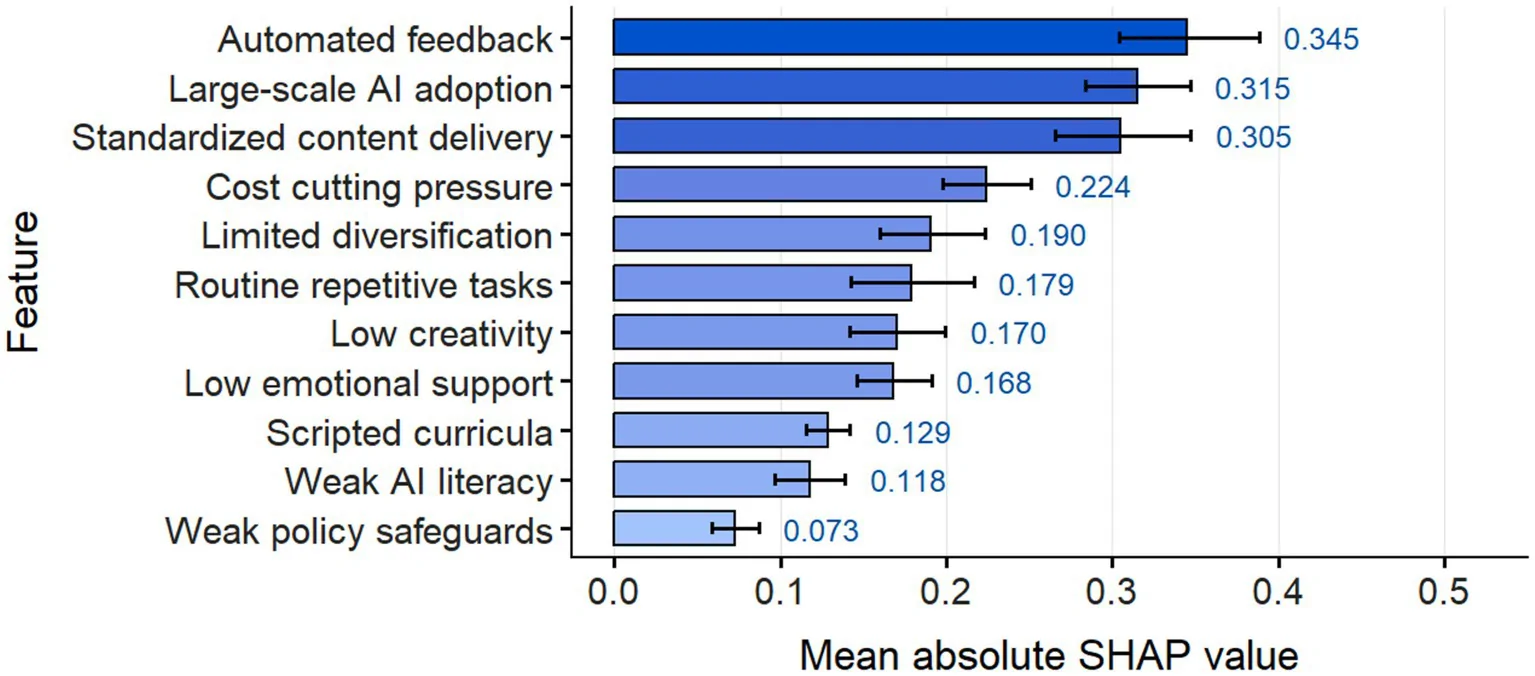
SHAP-based feature contributions to predicted teacher job-loss expectations in the held-out test. Bars show mean absolute SHAP values for each indicator, and error bars represent bootstrap 95% confidence intervals based on 2,000 resamples. Higher values indicate greater average contribution to the LightGBM model prediction, regardless of direction.

An additional SHAP directionality analysis was conducted by relating each predictor’s observed test-set values to its signed SHAP values. Across all 11 indicators, higher item ratings were associated with higher signed SHAP values, indicating that higher ratings on these institutional AI-related risk conditions were generally associated with more positive SHAP contributions and, consequently, higher model-predicted teacher job-loss expectation scores.

## 4 Discussion

This study examined whether the perceived current presence of AI-related risk conditions can predict stakeholder-estimated AI-related teacher job-loss expectations over the next decade, and which specific conditions carry the greatest predictive weight. Three research questions structured this inquiry: the descriptive and reliability properties of the institutional AI-related risk-condition indicators, the predictive capacity of these indicators for perceived teacher job-loss expectations, and the relative contribution of individual indicators to that prediction.

Regarding the first research question, the descriptive and reliability findings indicate that the 11 indicators provided a coherent and interpretable set of perceived institutional AI-related risk conditions. Internal consistency was good, suggesting that the indicators were sufficiently related to be interpreted as a thematically coherent set, while still representing distinct institutional conditions. At the same time, the descriptive statistics showed that these conditions were not perceived as uniformly or strongly established. Instead, the item ratings pointed to an uneven and emerging institutional landscape. This interpretation is consistent with evidence that AI use in education is expanding unevenly and often ahead of institutional guidance (Kaufman et al., 2025). The most visible conditions were weak AI literacy, weak policy safeguards, automated feedback, routine task automation, and large-scale AI adoption, whereas scripted curricula, low emotional support, and low recognition of creativity and professional judgment were less strongly endorsed. This pattern suggests that participants perceived AI-related institutional change as already visible in areas connected to guidance, preparation, feedback, routine work, and platform adoption. Evidence also shows that AI is already entering routine teacher work, including lesson planning, quiz generation, feedback, assessment, and administrative support (Borgonovi et al., 2025; OECD, 2026). Moreover, studies of AI-assisted grading and feedback suggest that teachers are experimenting with automated feedback and AI-supported assessment while continuing to emphasize the need for human oversight (Henderson et al., 2025). However, this change does not yet appear to represent a fully established displacement-oriented transformation of teaching. This conclusion is also supported by the present study’s relatively low-to-moderate item ratings and job-loss expectation scores. In support of this, a recent survey found that only 18% of United States K–12 teachers reported receiving formal guidance on AI use (Ash and Senseman, 2026).

Regarding the second research question, the machine-learning findings show that the extent to which stakeholders perceived current institutional AI-related risk conditions to be present had meaningful predictive value for the extent of teacher job loss they expected over the next 10 years. The model explained 68.3% of the variance in the original held-out test set and approximately 60% on average across 100 repeated held-out train-test splits, indicating good and stable predictive performance for a perception-based, cross-sectional model. This suggests that participants’ expectations about possible future loss of teaching positions were not random or disconnected from their perceptions of current institutional conditions. Rather, the indicators contained a substantial predictive signal: when stakeholders perceived stronger AI-related institutional risk conditions, the model was able to estimate higher teacher job-loss expectation scores with reasonable accuracy. This interpretation aligns with studies showing that teachers’ responses to AI are associated with institutional support, AI understanding, confidence, perceived benefits, and concerns about professional change (Sibug et al., 2025; Verano-Tacoronte et al., 2025; Viberg et al., 2025). It also agrees with broader automation research showing that awareness of job-automating technologies is associated with perceived job insecurity, especially when organizational conditions make workers feel less protected or less involved in technological change (Brougham and Haar, 2018; Li et al., 2019).

Regarding the third research question, SHAP was used to estimate how much each indicator contributed to the machine learning model’s predictions of teacher job-loss expectations. These results are best interpreted in contribution bands rather than as a strict rank order. The highest-contribution band included automated feedback, large-scale AI adoption, and standardized content delivery. Together, these predictors made the largest average contributions to the model’s predictions of teacher job-loss expectation scores. This corroborates evidence that AI is increasingly used for assessment, feedback, lesson planning, and workload reduction (Denny et al., 2024; OECD, 2026), and with concerns that platformization, datafication, and algorithmic management can shift pedagogical authority from teachers to platforms and dashboards (Kellogg et al., 2020; Knox et al., 2020; Parent-Rocheleau and Parker, 2022; Williamson et al., 2020). A secondary contribution band included cost-cutting pressure, limited role diversification, routine repetitive tasks, low creativity and judgment, and low emotional support. These predictors contributed less strongly than those in the top band, but they indicate that broader institutional pressures and the extent to which AI adoption expands or narrows teachers’ professional roles also contributed to the model’s predictions of perceived job-loss risk. This aligns with research showing that generative AI can either extend or constrain teacher agency depending on whether teachers retain control over design, interpretation, and pedagogical decision-making (Cukurova et al., 2025; Frøsig and Romero, 2024). The lower-contribution band included scripted curricula, weak AI literacy, and weak policy safeguards. These indicators should not be viewed as unimportant; rather, they may operate as background vulnerabilities, while direct indicators of instructional automation and platform adoption function as stronger signals of potential teacher job loss. UNESCO’s guidance similarly emphasizes that teacher competencies and AI governance remain essential for responsible AI integration and human agency (Miao and Cukurova, 2024; UNESCO, 2025).

Collectively, the study makes three closely connected contributions to research on AI and educational change. Methodologically, it demonstrates how held-out machine-learning prediction can be combined with SHAP-based interpretation to examine a perception-based outcome while maintaining transparency about the relative contribution of individual predictors. This approach extends the use of explainable machine learning in educational research beyond predictive accuracy alone by showing how complex predictive patterns can be interpreted in substantively meaningful terms. Empirically, the study identifies the institutional conditions that made the largest average contributions to the model’s predictions of teacher job-loss expectation scores. These findings provide evidence about which currently perceived institutional developments correspond most strongly with stakeholders’ expectations of possible teacher job loss. Theoretically, the study advances an institutional account of perceived teacher job-loss risk by conceptualizing such expectations as embedded in the ways AI is adopted, governed, and integrated into educational work. This perspective shifts attention away from technological capability alone and toward the wider institutional context in which AI-mediated changes to teaching are interpreted.

The practical implications of these findings are that educational institutions should treat AI integration not simply as a technical upgrade, but as a governance, pedagogy, and workforce-design issue. Because automated feedback, large-scale AI platform adoption, and standardized content delivery were the strongest predictors of perceived teacher job-loss expectations, institutions should clarify where AI is intended to support teachers and where it risks substituting for core pedagogical work, particularly in assessment, feedback, lesson planning, and learning analytics. This is especially important because poorly governed AI adoption may reproduce the risks identified in a potential collapse scenario in education, including over-automation, opaque decision-making, weakened teacher agency, job hollowing, inequitable access, and the erosion of trust in educational systems (Georgiou, 2026b). International guidance emphasizes that AI use in education should remain human-centered, transparent, accountable, and subject to professional oversight (UNESCO, 2019). Teacher professional development should therefore move beyond basic tool use and develop critical AI literacy, including the ability to evaluate bias, validity, privacy, transparency, and pedagogical appropriateness (Akgun and Greenhow, 2022; Celik, 2023). Institutions should also create expanded AI-related teacher roles, such as feedback moderator, AI curriculum designer, learning-data interpreter, and ethics reviewer, so that AI adoption becomes a pathway for professional renewal rather than job loss. Finally, implementation policies should explicitly protect the relational, ethical, creative, and contextual dimensions of teaching, since these remain central to educational quality and are not easily reducible to automated or standardized platform functions (Chan and Tsi, 2024; Seo et al., 2021).

## 5 Conclusion

This study provides three main findings in response to the research questions. First, AI-related teacher job-loss expectations are not reducible to isolated technologies or individual AI tools; rather, they are embedded in a wider institutional ecology involving automation, platform adoption, governance capacity, cost pressures, professional agency, and the value placed on human teaching. Second, the machine-learning prediction shows that perceived current institutional AI-related conditions are meaningfully linked with perceived future teacher job-loss expectations, indicating that stakeholders’ views of future risk correspond to what they already observe in educational settings today. Third, the strongest contribution band consisted of automated feedback, large-scale AI adoption, and standardized content delivery, indicating that signs of AI becoming embedded in core instructional functions and institutional platforms made the largest average contributions to the model’s predictions of job-loss expectation scores. Taken together, these findings suggest that perceived AI-related teacher job-loss risk is not viewed as an inevitable technological outcome but is associated with stakeholders’ perceptions of how education systems organize, govern, and implement AI.

Several limitations should be acknowledged. First, the study measured stakeholder expectations of future teacher job loss rather than actual employment outcomes, staffing reductions, or longitudinal changes in teachers’ work. Therefore, the model’s predictive performance should be interpreted as evidence of meaningful perceived risk, not as a direct forecast of future teacher employment. Second, because the predictors and outcome were rated by the same respondents within the same questionnaire, part of the observed association may reflect concurrent shared-perception variance or a general evaluative tendency toward AI risk. The present design cannot fully separate this common perceptual factor from substantive associations between specific institutional conditions and teacher job-loss expectations. Third, the teacher job-loss expectation outcome was measured using a single global 0–10 item. Although this format was appropriate for capturing an overall expectancy judgment, it did not permit the assessment of internal consistency or other forms of multi-item reliability and may offer less measurement precision than a validated multi-item measure. It also does not capture the different dimensions or mechanisms of AI-related teacher labor change. Fourth, although the sample included several relevant stakeholder groups, it was mainly drawn from Cyprus, which may limit generalizability to education systems with different labor protections, AI policies, funding pressures, technological infrastructures, and cultural expectations about teaching. Fifth, the cross-sectional design does not permit causal conclusions about whether institutional AI-related risk conditions lead to stronger job-loss expectations.

Future studies should therefore test the indicators in larger and more internationally diverse samples, examine their stability across stakeholder groups and educational sectors, and use longitudinal designs to determine whether changes in institutional AI adoption predict changes in teacher autonomy, workload, staffing patterns, and professional identity over time. Further research should also combine predictive modeling with qualitative interviews, classroom observations, policy analysis, and institutional case studies to explain how AI is actually reorganizing teaching work in practice. Such work would help identify the conditions under which AI becomes a source of displacement, augmentation, or professional renewal for teachers.

## Data Availability

The raw data supporting the conclusions of this article will be made available by the authors, without undue reservation.
